# Twelve-month follow-up of a randomised clinical trial of a brief group psychological intervention for common mental disorders in Syrian refugees in Jordan

**DOI:** 10.1017/S2045796022000658

**Published:** 2022-11-15

**Authors:** Richard A. Bryant, Ahmad Bawaneh, Manar Awwad, Hadeel Al-Hayek, Luana Giardinelli, Claire Whitney, Mark J. D. Jordans, Pim Cuijpers, Marit Sijbrandij, Peter Ventevogel, Katie Dawson, Aemal Akhtar

**Affiliations:** 1 School of Psychology, University of New South Wales, Sydney, Australia; 2 Brain Dynamics Centre, Westmead Institute of Medical Research, Sydney, Australia; 3 Jordan Country Office, International Medical Corps, Amman, Jordan; 4 Technical Unit, International Medical Corps, Washington, DC, USA; 5 Research and Development Department, War Child, Amsterdam, Netherlands; 6 Amsterdam Institute of Social Science Research, University of Amsterdam, Amsterdam, Netherlands; 7 Department of Clinical, Neuro and Developmental Psychology and WHO Collaborating Center for Research and Dissemination of Psychological Interventions, Vrije Universiteit, Amsterdam, Netherlands; 8 United Nations High Commissioner for Refugees, Geneva, Switzerland

**Keywords:** Anxiety, controlled trial, depression, psychological treatment, refugees, scalable intervention

## Abstract

**Aims:**

There is increasing evidence that brief psychological interventions delivered by lay providers can reduce common mental disorders in the short-term. This study evaluates the longer-term impact of a brief, lay provider delivered group psychological intervention (Group Problem Management Plus; gPM+) on the mental health of refugees and their children's mental health.

**Methods:**

This single-blind, parallel, controlled trial randomised 410 adult Syrians in Azraq Refugee Camp in Jordan who screened positive for distress and impaired functioning to either five sessions of gPM+ or enhanced usual care (EUC). Primary outcomes were scores on the Hopkins Symptom Checklist-25 (HSCL-25; depression and anxiety scales) assessed at baseline, 6 weeks, 3 months and 12 months Secondary outcomes included disability, posttraumatic stress, personally identified problems, prolonged grief, prodromal psychotic symptoms, parenting behaviour and children's mental health.

**Results:**

Between 15 October 2019 and 2 March 2020, 204 participants were assigned to gPM + and 206 to EUC, and 307 (74.9%) were retained at 12 months. Intent-to-treat analyses indicated that although participants in gPM + had greater reductions in depression at 3 months, at 12 months there were no significant differences between treatment arms on depression (mean difference −0.9, 95% CI −3.2 to 1.3; *p* = 0.39) or anxiety (mean difference −1.7, 95% CI −4.8 to −1.3; *p* = 0.06). There were no significant differences between conditions for secondary outcomes except that participants in gPM + had greater increases in positive parenting.

**Conclusions:**

The short-term benefits of a brief, psychological programme delivered by lay providers may not be sustained over longer time periods, and there is a need for sustainable programmes that can prolong benefits gained through gPM + .

A major challenge facing health systems in low-and-middle-income countries (LMICs) is how to mitigate common mental disorders in settings where health systems are not adequately resourced to provide psychological services, including insufficient numbers of mental health specialists. The Lancet Commission on Global Mental Health and Sustainable Development pointed to the gaps that exist between the need for mental health services and the capacity of LMICs to provide assistance (Patel *et al*., 2018) which is supported by much evidence (Moitra *et al*., 2022). This situation has led to task-sharing initiatives, where non-specialists receive brief trainings in psychosocial programmes to deliver mental health services in LMICs. Meta-analysis indicates that it can achieve a moderate effect size in alleviating common mental disorders, such as anxiety and depression (Singla *et al*., 2017). One widely used approach involves Problem Management Plus (PM + ). PM + was developed by the World Health Organization as a brief, transdiagnostic intervention to reduce common psychological disorders in people affected by adversity (WHO, 2016). This intervention teaches skills in arousal reduction, problem management, behavioural activation and accessing social support across five sessions (Dawson *et al*., 2015). PM + delivered in individual (Rahman *et al*., 2016; Bryant *et al*., 2017) and group (Rahman *et al*., 2019; Jordans *et al*., 2021; Bryant *et al.*, 2022) formats has been shown to be effective in reducing common mental disorders.

The evidence for PM + is limited, however, by relatively short-term follow-up assessments, typically with only three months follow-ups (Rahman *et al*., 2016, 2019; Bryant *et al*., 2017; Jordans *et al*., 2021). This seriously limits our understanding of the longer-term effects of brief psychological interventions in LMIC. Task-sharing interventions are typically implemented among populations exposed to ongoing stressors, including poverty, overcrowding, violence and other adversities, which is likely to increase psychological difficulties after completion of brief psychological interventions (Charlson *et al*., 2019). To address the lack of longer-term follow-up assessments in brief, task-sharing interventions, this study reports on a 12-month follow-up of a controlled trial of group PM + (gPM + ) that was conducted with Syrian refugees residing in a camp in Jordan (Akhtar *et al*., 2020) as part of multi-site research programme on scalable psychological interventions for Syrian refugees STRENGTHS Consortium (Sijbrandij *et al*., 2017). This trial aimed to assess the extent to which gPM + could reduce anxiety, depression, personally identified problems, unhelpful parenting behaviours and refugees' children psychological distress. Prior reporting on this study found that at the 3-month follow-up, refugees who received gPM + achieved greater reductions in the primary outcome of depression, as well as personally identified problems and inconsistent disciplinary parenting, relative to those who received enhanced usual care (EUC) (Bryant *et al*., 2022). We hypothesised that refugees receiving PM + would still have greater reductions in depression, personally identified problems and inconsistent disciplinary parenting at 12-months relative to those receiving EUC.

## Methods

### Trial design

This two-arm, single-blind randomised controlled trial was conducted in Azraq Refugee Camp in Jordan in partnership with the International Medical Corps (IMC) Jordan. The project was prospectively registered (Australian and New Zealand Clinical Trials Registry, no. 12619001386123) and the trial protocol is available in online Supplementary Information (S1 Protocol) and published (Akhtar *et al*., 2020). This study is reported as per the Consolidated Standards of Reporting Trials (CONSORT) guideline (online Supplementary Information S2 Checklist).

### Participants

The participants were refugees residing in Azraq Camp who were: (a) aged ⩾ 18 years, (b) Arabic-speaking, (c) scored ⩾16 on the Kessler Psychological Distress Scale (K10; Kessler *et al*., 2002), (d) scored ⩾17 on the WHO 12 item Disability Assessment Schedule 2.0 (WHODAS 2.0; WHODAS Group, 2000), and (e) had a child or dependent living in the household aged 10–16 years. The latter criterion was included to measure the secondary effects of gPM + on the mental health of participants' children. The K10 is a 10-item questionnaire that assesses psychological distress, with a range of 10–50, and the cut-off of 16 has been successfully used in refugee populations to identify distress (Shawyer *et al*., 2017). The WHODAS 2.0 is a 12-item questionnaire that assesses general functioning, with a possible total score of 48, and the cut-off followed prior trials of gPM + to ensure that participants were experiencing impaired function (Rahman *et al*., 2019). Participants were excluded if there was presence of: (a) significant cognitive or neurological impairment, (b) acute medical conditions, (c) severe mental disorders (e.g. psychotic or substance-abuse disorders) or (d) acute risk of suicide. Participants were recruited through door-to-door screening of consecutive caravans in the camp (one adult per caravan was invited). Participants provided informed consent for both the screening and, for those who screened positive, the trial. Caregivers also provided written consent for participation for one of their children to be assessed, from whom verbal assent was obtained.

Participants were randomly assigned on a 1:1 ratio to gPM + or EUC. Randomisation was conducted by staff at UNSW using software that generated random number sequences. Allocation concealment was maintained by assigning treatment conditions in sequentially numbered, sealed opaque envelopes that informed the trial co-ordinator in Jordan on assignment to gPM + or EUC. The assessors were blinded to group allocation.

The gPM + treatment is described in more detail elsewhere (Dawson *et al*., 2015) (see online Supplementary Information Table 1). Across five weekly 2 h group sessions (6–12 people in gender-specific groups) participants were taught psychoeducation, stress management focusing on breathing retraining, problem management, behavioural activation and accessing social support. Each group was led by two facilitators who had a bachelor's degree in social science or related discipline, spoke Arabic, but had limited to no prior experience in psychosocial programmes. Facilitators each received eight days of training that included group facilitation skills and gPM + delivery, followed by supervision during two practice cycles of gPM + . EUC comprised a 15-min visit to the participant's caravan by IMC staff who provided them with specific referral information of psychosocial services available in the camp that were appropriate for the types of problems identified in their baseline assessment (e.g. mental health, vocational training).

A local Study Safety Committee that comprised three Jordanian health professionals was established to monitor any adverse events that occurred during the trial. All adverse events were reported by assessors or gPM + facilitators to the committee, who referred to local services.

### Outcomes

All outcome measures underwent cultural adaptation and where necessary were translated and back-translated into Arabic. Full details of the adaptation process are published elsewhere (Akhtar *et al*., 2020, 2021*b*). The primary outcomes were total scores on the depression and anxiety scales of the 25-item Hopkins Symptom Checklist-25 (HSCL-25). The HSCL-25 has been validated in Arabic contexts, where recommended cut-offs for probable caseness of anxiety and depression relative to structured clinical interview is 2.0 and 2.1, respectively (Mahfoud *et al*., 2013). The internal consistency of the HSCL-25 in the current sample was robust for the anxiety (0.79) and depression (0.84) scales. The secondary outcomes assessed disability with the WHODAS 2.0 because it is important to gauge functional outcomes as distinct from psychopathology outcomes; posttraumatic stress disorder (PTSD) symptoms with the PTSD Checklist for DSM-5 (PCL-5; Blevins *et al*., 2015) because Syrian refugees have elevated rates of PTSD (Nguyen *et al*., 2022); personally identified problems with the Psychological Outcome Profiles (PSYCHLOPS; Ashworth, 2004) to obtain an index of problems that are not captured by standardised measures; grief with the PG-13 (Prigerson *et al*., 2009) because of the high rates of prolonged grief among Syrian refugees (Bryant *et al*., 2021); prodromal psychosis symptoms with the Prodromal Questionnaire-16 (PQ-B; Loewy *et al*., 2011) because refugees are at heightened risk for psychosis (Brandt *et al*., 2019); parenting skills with the Alabama Parenting Questionnaire-42 (APQ; Maguin *et al*., 2016) that measures parental involvement, poor supervision, positive parenting, inconsistent discipline and corporal punishment to determine if gPM + impacted refugee's parenting; and refugees' children's mental health with the Paediatric Symptom Checklist (Jellinek *et al*., 1999) that comprises internalising, externalising and attentional problem subscales. At baseline and 12 months, participants completed an adapted 27-item Traumatic Events Checklists (Shoeb *et al*., 2007) to measure exposure to traumatic events and a 17-item Post-Migration Living Difficulties checklist (Silove *et al*., 1997) to assess post-migration challenges.

Assessments were administered by Arabic-speaking Jordanians, who received four days of training in the assessment battery and psychological first aid. Assessors administered each assessment verbally and entered responses on portable tablets. All participants were given a gift worth 3 JOD ($US4) to reimburse their time for completing the post-intervention, 3-month, and 12-month assessments but were not reimbursed for participants in the gPM + sessions.

### Statistical analyses

The initial power analysis estimated that a medium effect size (*d* = 0.4) for gPM + could be achieved at the 3-month primary follow-up through a sample size of 133 participants per group (power = 0.90, *a* = 0.05, two-sided); the trial estimated that there would be 35% attrition at 3 months follow-up, and so the trial included 410 participants (205 in gPM + and 205 in EUC).

## Results

**Fig. 1.**
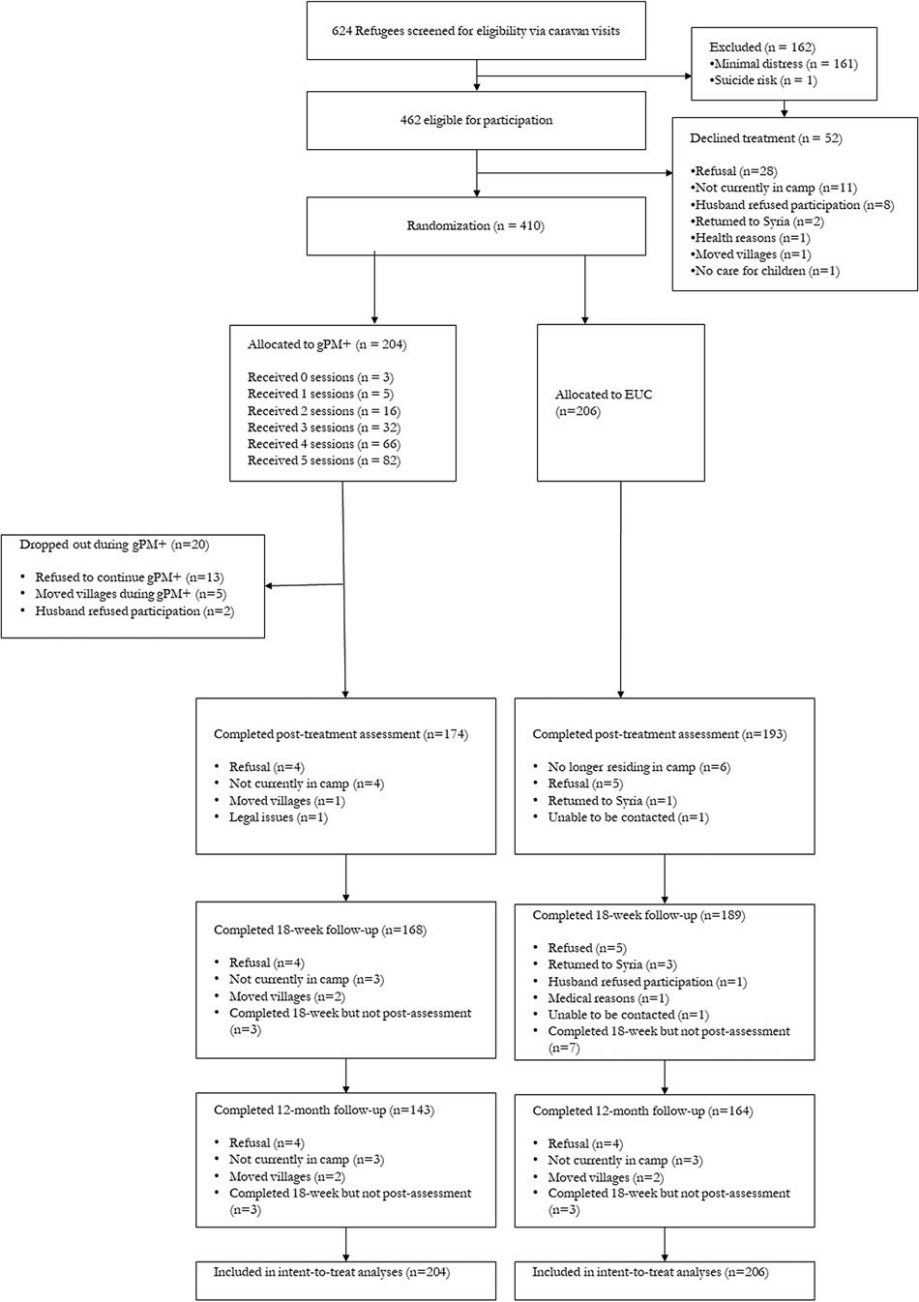
Flow diagram of progress through phases of a randomised trial comparing the group problem management plus intervention *v.* EUC in Syrian refugees in Azraq Refugee Camp, Jordan.

Participants were enroled between 14 October 2019 and 2 March 2020, and the final 12-month assessments were conducted on 31 May 2021. There were 1377 caravans approached during screen, 624 refugees agreed to be screened, of which 462 met entry criteria and 410 proceeded to randomisation (204 randomised to gPM + and 206 to EUC). Full details of the participants are reported in the prior report of the study (Bryant *et al*., 2022) and reported in online Supplementary Tables 2 and 3. The mean age of participants was 40.03 years (s.d. 6.95), most were female (70.2% females) and the mean time since leaving their home in Syria was 5.89 years (s.d. 1.67; range: 1–9 years). The most common living difficulties at 12 months were poverty (86.7%), difficulties with employment (89.5%), worry about family (65.5%) and fear of being returned to Syria (64.3%) (see Table 1). The demographic characteristics were not significantly different between the two treatment arms. The 12-month assessment was conducted for 307 participants (74.9%) participants. There were more participants retained in the EUC condition (164, 79.6%) than gPM + (143, 70.1%), *χ*^2^ = 4.9, *p* = 0.03. Participants who did and did not complete the 12-month assessment did not differ on any baseline characteristics (with a Bonferroni-adjusted *ɑ* = 0.002), however those who dropped out had marginally higher depression and PTSD scores than those who were retained (Table 2). The flowchart of participant recruitment and retention is reported in Fig. 1. Although the number of postmigration stressors reported at baseline (26.0 ± 11.0) was higher than reported at 12 months (17.2 ± 7.7), the ongoing stressors were still at a high level. No adverse events were attributable to the interventions or the trial.

**Table 1.** Frequencies and percentages of rates of post-migration living difficulties at 12 months

Current stressors *N* (%)	Total (*n* = 307)	gPM + (*n* = 117)	Enhanced usual care (*n* = 131)
Communication difficulties	59 (19.2)	26 (18.2)	33 (20.1)
Discrimination	73 (23.8)	35 (24.5)	38 (23.2)
Ethnic conflict	25 (8.1)	11 (7.7)	14 (8.5)
Family separation	117 (38.1)	64 (44.8)	53 (32.3)
Worry for family	201 (65.5)	92 (64.3)	109 (66.5)
Unable to return home	41 (28.7)	60 (36.6)	101 (32.9)
Difficulties with employment	128 (89.5)	144 (88)	272 (88.6)
Difficulties with immigration officials	0 (0.0)	2 (1.2)	2 (0.7)
Difficulties with other officials	1 (0.7)	2 (1.2)	3 (1.0)
Not recognised as a refugee	0 (0.0)	0 (0.0)	0 (0.0)
Fear of being returned to Syria	92 (64.3)	93 (56.7)	185 (60.3)
Lack of healthcare	55 (38.5)	65 (39.6)	120 (39.1)
Poverty	124 (86.7)	126 (76.8)	250 (81.4)
Difficulties with financial assistance	66 (46.2)	82 (50.0)	148 (48.2)
Loneliness	57 (39.9)	51 (31.1)	108 (35.2)
Language difficulties	0 (0.0)	0 (0.0)	0 (0.0)
Accommodation difficulties	3 (2.1)	0 (0.0)	3 (1.0)

**Table 2.** Participant characteristics

	Completed 12-month assessment (*n* = 307)	Not completed 12-month assessmen (*n* = 103)	*T/χ*^2^	*p*
Female, *n* (%)	225 (73.3)	75 (72.8)	0.01	
Age, years (s.d.)	40.1 (7.2)	39.9 (6.2)	0.27	0.79
Time since leaving Syria, y	5.9 (1.6)	5.9 (1.7)	−0.09	0.93
Number of traumatic events	7.45 (3.9)	8.3 (4.5)	−1.90	0.06
Number of postmigration stressors	25.9 (10.8)	26.5 (11.5)	−0.53	0.60
Married, *n* (%)			4.24	
Single	0 (0)	0 (0)		
Married	281 (91.5)	95 (92.2)		
Separated	13 (4.2)	3 (2.9)		
Divorced	2 (0.7)	3 (2.9)		
Widowed	11 (3.6)	2 (1.9)		
Education, *n* (%)			2.93	
None	73 (23.8)	29 (28.2)		
Basic certificate (10 years of eduction)	178 (58.0)	55 (53.4)		
Technical trade certificate	25 (8.1)	12 (11.7)		
Secondary education (12 years of education)	24 (7.8)	5 (4.9)		
University degree	7 (2.3)	2 (1.9)		
Baseline HSCL: depression	35.2 (8.8)	37.7 (9.5)	−2.44	0.02
Baseline HSCL: anxiety	24.6 (6.2)	25.9 (6.0)	−1.87	0.06
Baseline PCL-5	25.5 (14.5)	28.9 (14.6)	−2.06	0.04
Baseline WHODAS	23.5 (4.8)	24.3 (5.5)	−1.43	0.15
Baseline PSYCHLOPS	16.0 (3.9)	16.5 (3.8)	−1.15	0.25
Baseline PG13	28.5 (10.0)	28.7 (10.1)	−0.19	0.85
Baseline PQ-B	13.4 (2.9)	13.0 (3.2)	1.21	0.23
APQ: parental involvement	34.4 (2.9)	35.7 (8.3)	−1.31	0.19
APQ: positive parenting	24.1 (5.1)	25.0(4.4)	−1.57	0.12
APQ: poor supervision	14.7 (4.6)	15.6 (5.3)	−1.68	0.09
APQ: inconsistent discipline	15.1(4.0)	15.3 (4.2)	−0.50	0.62
APQ: punishment	6.2 (2.7)	6.2 (2.8)	−0.13	0.89
PSC: internalising problems	8.3 (1.5)	8.3 (1.6)	−0.28	0.78
PSC: externalising problems	10.6 (1.6)	10.6 (1.6)	−0.21	0.84
PSC: attentional problems	9.3 (2.3)	8.9 (2.1)	1.40	0.16

gPM + , Group Problem Management Plus; EUC, Enhanced usual care; HSCL, Hopkins Symptom Checklist (depression subscale score range: 10–40; anxiety subscale score range: 15–60; higher scores indicate elevated anxiety or depression); WHODAS, WHO Disability Assessment Schedule (total score range: 0–48; higher scores indicate more severe impairment); PCL-5, Posttraumatic Stress Disorder Checklist (total score range: 0–80; higher scores indicate more severe PTSD severity); PSYCHLOPS, Psychological Outcomes Profiles (total score range: 0–20; higher scores indicate poorer outcome); PG-13, Prolonged Grief Disorder 13 (total score range: 11–57; higher scores indicate poorer outcome); PQ, Prodromal Questionnaire (total score range: 0–64; higher scores indicate poorer outcome). Alabama Parenting Questionnaire (Parental Involvement subscale score range: 10–50; Positive Parent subscale score range: 6–30; Supervision subscale score range 10–50; Discipline subscale score range 6–30; Punishment subscale score range 3–15; higher scores indicate elevated parental involvement, positive parenting, supervision, discipline and punishment). Paediatric Symptom Checklist is child's self-report (PSC; Attention Problems subscale score range: 0–10; Internalising subscale score range: 0–10; Externalising subscale score range: 0–14).

Regarding primary outcomes, at the 12-month assessment there were no significant differences between gPM + and EUC on either HSCL-Depression (adjusted mean difference −1.0, 95% CI −3.2 to 1.3; *p* = 0.39; effect size, 0.11) or HSCL-Anxiety (adjusted mean difference −1.7, 95% CI −4.8 to −1.3; *p* = 0.06; effect size, 0.27) scores (Table 3). It should be noted that although there were no significant differences between these conditions, participants in EUC tended to have greater reductions at 12 months relative to gPM + , and especially in terms of anxiety. There were no differences between the two treatment arms at 12-months on any of the secondary outcomes, with the exception that participants in gPM + reported more improved positive parenting relative to those in EUC (adjusted mean difference −2.0, 95% CI −3.7 to −0.3; *p* = 0.02; effect size, 0.41) (Table 3).

**Table 3.** Summary statistics and results from mixed model analysis of primary and secondary outcomes

		Descriptive statistics		Mixed model analysis		
Primary and secondary outcomes	Visit	gPM + (*n* = 168)	EUC (*n* = 189)	Difference in LS mean (95% CI)	*p*-value	Effect size
		Estimated mean (s.e.)	Estimated mean (s.e.)			
HSCL-25 Depression	Baseline	36.57 (0.67)	35.15 (0.67)			
	6-week	29.95 (0.72)	32.44 (0.69)	3.92 (1.84–5.99)	0.001	0.43
	3 months	29.03 (0.73)	31.28 (0.69)	3.68 (1.54–5.81)	0.001	0.40
	12 months	28.26 (0.78)	25.85 (0.73)	−0.98 (−3.23 to 1.26)	0.39	−0.11
HSCL-25 Anxiety	Baseline	24.82 (0.46)	25.00 (0.46)			
	6-week	20.42 (0.49)	21.95 (0.47)	1.35 (−0.22 to 2.93)	0.09	0.22
	3 months	20.03 (0.50)	19.64 (0.47)	−0.57 (−2.21 to 1.06)	0.49	−0.09
	12 months	19.34 (0.54)	17.85 (0.50)	−1.67 (−3.39 to 0.04)	0.06	−0.27
WHODAS 2.0	Baseline	23.53 (0.47)	23.92 (0.47)			
	6-week	14.70 (0.49)	15.63 (0.51)	0.87 (−1.4 to 2.22)	0.53	0.17
	3 months	15.73 (0.50)	14.98 (0.51)	−1.14 (−2.96 to 0.69)	0.22	−0.23
	12 months	15.16 (0.56)	13.81 (0.53)	−1.74 (−3.67 to 0.19)	0.08	−0.35
PCL-5	Baseline	25.98 (0.91)	26.72 (0.90)			
	6-week	16.07 (0.98)	17.54 (0.93)	0.73 (−2.52 to 3.98)	0.66	0.05
	3 months	10.29 (0.99)	10.32 (0.94)	0.01 (−3.97 to 2.54)	0.67	0.00
	12 months	2.60 (1.07)	3.29 (1.00)	−0.06 (−3.48 to 3.36)	0.97	0.00
PSYCHLOPS	Baseline	16.48 (0.31)	15.72 (0.31)			
	6-week	13.35 (0.33)	13.68 (0.32)	1.08 (0.18–1.99)	0.02	0.28
	3 months	13.49 (0.34)	13.60 (0.32)	0.87 (−0.11 to 1.84)	0.08	0.22
	12 months	12.45 (0.36)	11.77 (0.34)	0.07 (−0.95 to 1.10)	0.89	0.02
PG-13	Baseline	27.97 (0.85)	29.19 (0.87)			
	6-week	26.83 (0.90)	27.73 (0.91)	−0.32 (−3.06 to 2.43)	0.82	−0.03
	3 months	20.48 (0.92)	21.42 (0.91)	−0.28 (−3.20 to 2.63)	0.85	−0.03
	12 months	19.71 (0.99)	19.79 (0.99)	−1.15 (−4.26 to 1.96)	0.47	−0.12
PQ-B	Baseline	13.33 (0.15)	13.41 (0.15)			
	6-week	14.84 (0.16)	14.44 (0.16)	−0.53 (−1.10 to 0.04)	0.07	−0.18
	3 months	15.07 (0.17)	14.96 (0.16)	−0.24 (−0.82 to 0.33)	0.41	−0.08
	12 months	15.88 (0.18)	15.79 (0.17)	−0.19 (−0.81 to 0.43)	0.55	−0.07
APQ: Involvement	Baseline	34.96 (0.82)	34.56 (0.82)			
	6-week	33.75 (0.88)	32.95 (0.85)	−0.39 (−3.46 to 2.67)	0.80	−0.04
	3 months	31.90 0.90)	31.86 (0.85)	0.37 (−2.898 to 3.62)	0.83	0.04
	12 months	34.41 0.90)	37.26 (0.91)	3.25 (−0.08 to 6.58)	0.06	0.38
APQ: Supervision	Baseline	14.97 (0.34)	14.81 (0.34)			
	6-week	12.91 (0.37)	13.54 (0.35)	0.78 (−0.46 to 2.02)	0.21	0.16
	3 months	12.42 (0.38)	12.42 (0.36)	0.16 (−1.188 to 1.50)	0.82	0.03
	12 months	13.34 (0.41)	12.23 (0.38)	−0.95 (−2.35 to 4.64)	0.19	−0.20
APQ: Positive parenting	Baseline	24.08 (0.43)	24.62 (0.42)			
	6-week	23.45 (0.46)	23.59 (0.44)	−0.40 (−1.949 to 1.14)	0.61	0.08
	3 months	21.86 (0.47)	22.26 (0.45)	−0.14 (−1.76 to 1.48)	0.86	−0.03
	12 months	24.19 (0.51)	22.73 (0.47)	−2.01 (−3.70 to −0.31)	0.02	−0.41
APQ: Discipline	Baseline	15.49 (0.32)	14.74 (0.32)			
	6-week	13.73 (0.34)	13.52 (0.33)	0.54 (−0.70 to 1.79)	0.39	0.14
	3 months	13.00 (0.35)	13.57 (0.33)	1.33 (0.17–2.49)	0.03	0.33
	12 months	13.29 (0.38)	12.36 (0.35)	−0.17 (−1.40 to 1.05)	0.78	−0.04
APQ: Punishment	Baseline	6.08 (0.15)	6.34 (0.15)			
	6-week	5.49 (0.16)	5.55 (0.16)	−0.09 (−0.71 to 0.31)	0.44	−0.03
	3 months	5.41 (0.17)	5.47 (0.16)	−0.27 (−0.79 to 0.31)	0.31	−0.10
	12 months	5.54 (0.18)	5.34 (0.17)	−0.46 (−1.01 to 0.13)	0.10	−0.17
PSC: Total score	Baseline	14.79 (0.49)	16.22 (0.48)			
	6-week	13.01 (0.54)	13.99 (0.52)	−0.46 (−2.09 to 1.19)	0.59	−0.06
	3 months	12.43 (0.53)	13.35 (0.53)	−0.52 (−2.23 to 1.20)	0.56	−0.08
	12 months	16.84 (0.62)	17.28 (0.56)	−1.00 (−2.87 to 0.87)	0.30	−0.14
PSC: Attention problems	Baseline	3.89 (0.16)	4.45 (0.16)			
	6-week	3.42 (0.17)	3.83 (0.17)	−0.22 (−0.77 to 0.34)	0.44	−0.10
	3 months	3.14 (0.18)	3.77 (0.17)	−0.23 (−0.82 to 0.37)	0.46	−0.10
	12 months	2.05 (0.20)	3.48 (0.17)	−0.35 (−1.00 to 0.30)	0.30	−0.16
PSC: Internalising	Baseline	3.22 (0.11)	3.35 (0.11)			
	6-week	2.84 (0.12)	2.97 (0.12)	0.01 (−0.38 to 0.40)	0.96	0.00
	3 months	2.85 (0.12)	3.01 (0.12)	0.04 (−0.38 to 0.45)	0.87	0.03
	12 months	2.66 (0.14)	2.63 (0.13)	−0.16 (−0.61 to 0.29)	0.49	−0.10
PSC: Externalising	Baseline	3.63 (0.11)	3.64 (0.11)			
	6-week	3.30 (0.12)	3.30 (0.12)	−0.01 (−0.43 to 0.41)	0.96	0.00
	3 months	3.20 (0.12)	3.37 (0.12)	0.17 (−0.25 to 0.59)	0.43	0.11
	12 months	3.04 (0.14)	2.88 (0.13)	−0.17 (−0.62 to 0.28)	0.46	−0.11

EUC, Enhanced usual care; LS, Least Square; HSCL, Hopkins Symptom Checklist (depression subscale score range: 10–40; anxiety subscale score range: 15–60; higher scores indicate elevated anxiety or depression); PQ-B, Brief Prodromal Questionnaire; WHODAS 2.0, WHO Disability Assessment Schedule 2.0 (total score range: 0–48; higher scores indicate more severe impairment); PCL-5, Posttraumatic Stress Disorder Checklist (total score range: 0–80; higher scores indicate more severe PTSD severity); PSYCHLOPS, Psychological Outcomes Profiles (total score range: 0–20; higher scores indicate poorer outcome); PG-13, Prolonged Grief Disorder 13 (total score range: 11–57; higher scores indicate poorer outcome); PQ, Prodromal Questionnaire; (total score range: 0–64; higher scores indicate poorer outcome); APQ, Alabama Parenting Questionnaire (Parental Involvement subscale score range: 10–50; Positive Parent subscale score range (total score range : 6–30; higher scores indicate poorer outcome); Supervision subscale score range 10–50; Discipline subscale score range 6–30; Punishment subscale score range 3–15; higher scores indicate elevated parental involvement, positive parenting, supervision, discipline and punishment). Paediatric Symptom Checklist is child's self-report (PSC; Attention Problems subscale score range: 0–10; Internalising subscale score range: 0–10; Externalising subscale score range: 0–14). Effect size was calculated by the difference in least square means between intervention and EUC from mixed model divided by the pooled standard deviation.

Supplementary analyses indicated that (a) focused only on participants who completed the 12-month assessment, and (b) controlled for ongoing stressors and exposure to traumatic events in the past 12 months also found no differences between treatment conditions (see online Supplementary Tables 4 and 5), except that after controlling for stressors and traumatic events gPM + resulted in greater changes in positive parenting relative to EUC (adjusted mean difference −2.44, 95% CI −4.53 to −0.36; *p* = 0.02; effect size, −0.50). We also conducted a secondary analysis that was not prescribed in the analysis plan to determine the proportion of participants whose depression and anxiety deteriorated between the 3- and 12-months assessments. Following previous methods of determining worsening of symptoms (Guidi *et al*., 2018), we calculated worsening of depression and anxiety with the minimally important difference by comparing the proportions of participants showing a reduction of more than 0.5 s.d.s of the depression and anxiety scores at the 12-month assessment relative to the 3-month scores (Norman *et al*., 2003). This analysis showed that comparable proportions of participants in gPM + (44, 30.8%) and EUC (38, 23.2%) worsened in terms of anxiety (*χ*^2^ = 2.3, *p* = 0.13). There were more participants in gPM + (42, 29.4%) and EUC (26, 15.9%) worsened in terms of depression (*χ*^2^ = 8.1, *p* = 0.004).

## Discussion

Contrary to our hypotheses, gPM + did not show any benefit on the primary outcomes at the 12-month follow-up assessment. This pattern is in contrast to the observation that at 3-months gPM + led to greater reductions in depression. Further, the previously reported benefits of gPM + in reducing personally identified problems and inconsistent disciplinary parenting at 3-months were not maintained at 12 months. The finding that gPM + did not have longer-term benefits is consistent with prior evidence from more intensive programmes that have also reported that benefits of psychological interventions do not consistently persist, despite initial remission of symptoms (Paykel, 2007; Brouwer *et al*., 2019). There is evidence that more intensive psychotherapies for depression can have lasting benefit at 12 months (Cuijpers *et al*., 2021), however the current findings raise questions about the longevity of initial gains made with brief interventions, such as gPM + in populations experiencing ongoing stressors.

Several trials of (g)PM + have reported significant mental health benefits 3 months after participants received the intervention (Rahman *et al*., 2016; Bryant *et al*., 2017; Rahman *et al*., 2019; Jordans *et al*., 2021; Bryant, 2022). This is the first report of a longer-term follow-up of people who have received individual or group PM + . Our findings suggest that initial gains achieved by this brief intervention may not be long-lasting. This pattern is underscored by the observation that depression severity worsened more frequently in gPM + than those in EUC. Equally it is possible that over the 12 months many participants in EUC achieved the reduction in depression initially made by gPM + participants in the first three months. That is, those refugees who received gPM + achieved a reduction in depression faster than those in EUC but both groups achieved comparable depression levels at 12 months. It perhaps is not surprising that the benefits of PM + did not persist at 12 months because the intervention delivered by non-specialists is meant to be brief in that it is only comprised of five group sessions and is low-intensity. The gains of this brief intervention may be particularly constrained in a population with a significant history of traumatic events and experiencing ongoing stressors in the refugee camp. One prior study of gPM + found that 31% of the treatment effect of gPM + was due to the use of PM + strategies (Jordans *et al*., 2021), and it is possible that in the context of ongoing stressors in the camp use of these strategies may have waned. We note that we lack data to support this possibility, however in light of the greater reduction in depressive symptoms at 3 months that was not maintained at 12 months, one possibility is that the skills acquired during gPM + were not retained in the 12 months after the programme. It is also worth noting that the period between the 3- and 12-month assessments refugees around the world were adversely affected by a range of stressors arising from the COVID-19 pandemic, including lockdowns, impairments in resettlement, social isolation and shortage of supplies (Liddell *et al*., 2021; Garrido *et al*., 2022); these stressors have also been documented in Azraq camp (Akhtar *et al*., 2021*a*, 2021*b*). Further, most of the sample had probable PTSD, anxiety or depression, and these conditions are typically addressed with more intensive treatment programmes. Many recipients of PM + still experienced psychological problems after the programme (Akhtar *et al*., 2022), and so it very well possible that these difficulties are exacerbated in the months after the gPM + , especially in the context of ongoing stressors.

It may be argued that one limitation of brief, lay-delivered interventions is that they do not have long-term benefits. However, there are possible means to address the apparent short-term benefits of gPM + . One potentially useful way to address this issue is by delivering augmentation strategies to maintain initial gains. Several options are available and need to be subjected to full investigation. First, booster sessions could be offered by lay providers to remind people of strategies taught in PM + , and to brain-storm how these can be applied to ongoing stressors; evidence that use of gPM + strategies wanes over time, and use of these strategies mediates symptom reduction after gPM + underscores the potential utility of booster sessions (Jordans *et al*., 2021). Provision of booster sessions has been shown to be effective in preventing relapse for a range of psychological problems, including anxiety and depression (Gearing *et al*., 2013; Bruijniks *et al*., 2020). Second, stepped care programmes may have potential to triage more severely distressed people to more intensive programmes, whilst those with moderate distress may benefit from further brief interventions. There is evidence that stepped care programmes can be successfully implemented in LMICs (Araya *et al*., 2003; Patel *et al*., 2010). These trials evaluated stepped care programmes relative to usual care, and so have not directly addressed whether they confer additive benefits over standard brief interventions, such as PM + . It is possible that provision of augmentation strategies following gPM + may also benefit participants who did not attend every group session because it can provide them with the opportunity to learn or reinforce strategies that may have not been adequately rehearsed during the initial gPM + programme.

It is interesting that 12 months after the intervention, refugees who received gPM + reported a greater increase in positive parenting relative to those in EUC. There is evidence that positive parenting is associated with better children's mental health, including in children in Arab contexts affected by war (Thabet *et al*., 2009). Further, parenting programmes that promote positive parental behaviours have been shown to be effective in improving adaptive parenting behaviours and children's mental health (Lindsay *et al*., 2011). Programmes that have targeted caregivers' parenting skills have also reported improvements in adaptive parenting behaviours in contexts comprising refugees and other Arab settings affected by humanitarian crises (Lakkis *et al*., 2020; Miller *et al*., 2020). Notably, gPM + does not directly instruct in parenting behaviours, however it is possible that the skills taught in this programme may have led to alterations in parenting that are reflected in greater positive parenting scores. Alternately, improving caregivers' mental health may improve their parenting behaviour, which is one interpretation of findings that gPM + leads to better mental health and parenting (Bryant, 2022). We emphasise, however, that in the context of the primary outcomes and all other secondary outcomes being non-significant, this observation of greater positive parenting associated with gPM + may be a spurious finding and needs to be replicated before one can draw firm conclusions.

One of the curious findings of the 12-month follow-up was that PTSD severity reduced markedly in both the gPM + and EUC conditions. Several explanations may be offered for this pattern. First, between the 3-month and 12-month assessments the camp was directly affected by the COVID-19 pandemic, resulting in lockdowns in the camp that often restricted people to their caravans, which could limit participants' exposure to trauma reminders that can activate trauma memories and other PTSD symptoms. Second, the restrictions during the pandemic may have led to participants feeling greater safety because of the restriction to their own caravans. Third, the impact of the pandemic and the associated restrictions on all residents in the camp may have resulted in those participants with PTSD symptoms re-evaluating their own psychological health, and this may have led to a degree of normalisation of their PTSD symptoms; this interpretation accords with evidence during the pandemic that people with more severe psychological problems reported less severe psychological difficulties relative to their pre-pandemic states (Pan *et al*., 2021).

We note several methodological limitations of the study. First, retention at the 12-month assessment was 74.9% of the original sample, which raises the possibility that results may have been influenced by a biased sample. This concern is underscored by the observation that more participants in the EUC condition were retained at 12 months relative to gPM + , and that those who were retained tended to have higher depression and PTSD scores at baseline. The nature of those who were and were not retained at 12 months raises questions over the robustness of intent-to-treat analysis approach; although our secondary analyses that focused only on those who completed the 12-month follow-up, we acknowledge that the biased nature of the follow-up sample may limit the extent to which we can confidently draw conclusions from the final data. Second, not all of the measures were properly culturally adapted, which limit the appropriateness of these measures to assess the intended constructs. There is much evidence that cultural context can play an important role in how mental health is experienced and reported, and that measures developed in the west may not fully capture the nuances of Syrian refugees' psychological responses (Hinton and Lewis-Fernández, 2011). For example, the PSYCLOPS, PG-13, PQ-B and APQ have not been culturally or psychometrically validated in this population, and this can be problematic with constructs such as grief, prodromal psychotic signs and parenting domains. Third, we could not objectively measure the services accessed by the EUC participants. Fourth, the control condition did not match gPM + for weekly contact with a facilitator or other group members, and in this sense the design could not isolate the effects of gPM + relative to nonspecific effects. Recent evidence highlights that gains in brief interventions may be attributed to nonspecific effects (Riello *et al*., 2021), which highlights the need for control conditions that can dismantle the active ingredients of programmes. Finally, we recognise that these refugees had been in the camp for an average of five years and it is unknown how this lengthy period of displacement and detention may have impacted the longer-terms of gPM + . Further long-term follow-up assessments of refugee groups who have received gPM + and who have not been detained for such a lengthy period may yield different results.

Despite these limitations, the current findings suggest that the benefits of a brief, lay-provider intervention, such as gPM + , may not persist in the long-term. This conclusion does not undermine the utility of such interventions, which have been shown to effectively reduce common psychological disorders in people affected by adversity. The challenge for the field of global mental health is to develop ongoing programmes or service frameworks that can sustain the initial gains of effective interventions.

## Data Availability

The data that support the findings of this study are not publicly available until the STRENGTHS consortium has completed independent participant data analyses in association with related studies but will be available from Richard Bryant (r.bryant@unsw.edu.au) after these IPD analyses are complete.

## Supplementary material

For supplementary material accompanying this paper visit https://doi.org/10.1017/S2045796022000658. click here to view supplementary material

## References

[ref1] Akhtar A, Giardinelli L, Bawaneh A, Awwad M, Naser H, Whitney C, Jordans MJD, Sijbrandij M and Bryant RA (2020) Group problem management plus (gPM+) in the treatment of common mental disorders in Syrian refugees in a Jordanian camp: study protocol for a randomized controlled trial. BMC Public Health 20, 390. 32216762 10.1186/s12889-020-08463-5 PMC7098148

[ref2] Akhtar A, Bawaneh A, Awwad M, Al-Hayek H, Sijbrandij M, Cuijpers P and Bryant RA (2021 *a*) A longitudinal study of mental health before and during the COVID-19 pandemic in Syrian refugees. European Journal of Psychotraumatology 12, 1991651. 34777714 10.1080/20008198.2021.1991651 PMC8583939

[ref3] Akhtar A, Heidrun Engels MH, Bawaneh A, Bird M, Bryant R, Cuijpers P, Hansen P, Al-Hayek H, Ilkkursun Z, Kurt G, Sijbrandij M, Underhill J and Acarturk C (2021 *b*) Cultural adaptation of a low-intensity group psychological intervention for Syrian refugees. Intervention 9, 48–57.

[ref4] Akhtar A, Koyiet P, Rahman A, Schafer A, Hamdani SU, Cuijpers P, Sijbrandij M and Bryant RA (2022) Residual posttraumatic stress disorder symptoms after provision of brief behavioral intervention in low- and middle-income countries: an individual-patient data meta-analysis. Depression and Anxiety 39, 71–82. 34752690 10.1002/da.23221 PMC9299611

[ref5] Araya R, Rojas G, Fritsch R, Gaete J, Rojas M, Simon G and Peters TJ (2003) Treating depression in primary care in low-income women in Santiago, Chile: a randomised controlled trial. Lancet 361, 995–1000. 12660056 10.1016/S0140-6736(03)12825-5

[ref6] Ashworth M, Shepherd M, Christey J, Matthews V, Wright K, Parmentier H, Robinson S and Godfrey E (2004) A client-centred psychometric instrument: the development of ‘PSYCHLOPS’ (‘Psychological Outcome Profiles’). Counselling and Psychotherapy Research 4, 27–33.

[ref7] Blevins CA, Weathers FW, Davis MT, Witte TK and Domino JL (2015) The posttraumatic stress disorder checklist for DSM-5 (PCL-5): development and initial psychometric evaluation. Journal of Traumatic Stress 28, 489–498. 26606250 10.1002/jts.22059

[ref8] Brandt L, Henssler J, Muller M, Wall S, Gabel D and Heinz A (2019) Risk of psychosis among refugees: a systematic review and meta-analysis. JAMA Psychiatry 76, 1133–1140. 31411649 10.1001/jamapsychiatry.2019.1937 PMC6694397

[ref9] Brouwer ME, Williams AD, Kennis M, Fu Z, Klein NS, Cuijpers P and Bockting CL (2019) Psychological theories of depressive relapse and recurrence: a systematic review and meta-analysis of prospective studies. Clinical Psychology Review 74, 101773. 31756681 10.1016/j.cpr.2019.101773

[ref10] Bruijniks SJE, Lemmens L, Hollon SD, Peeters F, Cuijpers P, Arntz A, Dingemanse P, Willems L, van Oppen P, Twisk JWR, van den Boogaard M, Spijker J, Bosmans J and Huibers MJH (2020) The effects of once- versus twice-weekly sessions on psychotherapy outcomes in depressed patients. British Journal of Psychiatry 216, 222–230. 10.1192/bjp.2019.265 32029012

[ref11] Bryant RA, Schafer A, Dawson KS, Anjuri D, Mulili C, Ndogoni L, Koyiet P, Sijbrandij M, Ulate J, Harper Shehadeh M, Hadzi-Pavlovic D and van Ommeren M (2017) Effectiveness of a brief behavioural intervention on psychological distress among women with a history of gender-based violence in urban Kenya: a randomised clinical trial. PLoS Medicine 14, e1002371. 28809935 10.1371/journal.pmed.1002371 PMC5557357

[ref12] Bryant RA, Bawaneh A, Giardinelli L, Awwad M, Al-Hayek H and Akhtar A (2021) A prevalence assessment of prolonged grief disorder in Syrian refugees. World Psychiatry 20, 302–303. 34002523 10.1002/wps.20876 PMC8129832

[ref13] Bryant RA, Bawaneh A, Awwad M, Al-Hayek H, Giardinelli L, Whitney C, Jordans MJD, Cuijpers P, Sijbrandij M, Ventevogel P, Dawson K and Akhtar A (2022) Effectiveness of a brief group behavioral intervention for common mental disorders in Syrian refugees in Jordan: a randomized controlled trial. PLoS Medicine 19, e1003949. 35298469 10.1371/journal.pmed.1003949 PMC8929659

[ref14] Charlson F, van Ommeren M, Flaxman A, Cornett J, Whiteford H and Saxena S (2019) New WHO prevalence estimates of mental disorders in conflict settings: a systematic review and meta-analysis. Lancet 394, 240–248. 31200992 10.1016/S0140-6736(19)30934-1 PMC6657025

[ref15] Cuijpers P, Quero S, Noma H, Ciharova M, Miguel C, Karyotaki E, Cipriani A, Cristea IA and Furukawa TA (2021) Psychotherapies for depression: a network meta-analysis covering efficacy, acceptability and long-term outcomes of all main treatment types. World Psychiatry 20, 283–293. 34002502 10.1002/wps.20860 PMC8129869

[ref16] Dawson KS, Bryant RA, Harper M, Kuowei Tay A, Rahman A, Schafer A and van Ommeren M (2015) Problem management plus (PM + ): a WHO transdiagnostic psychological intervention for common mental health problems. World Psychiatry 14, 354–357. 26407793 10.1002/wps.20255 PMC4592660

[ref17] Garrido R, Paloma V, Benitez I, Skovdal M, Verelst A and Derluyn I (2022) Impact of COVID-19 pandemic on the psychological well-being of migrants and refugees settled in Spain. Ethnicity and Health, 1–24. doi: 10.1080/13557858.2022.2035692 35138212

[ref18] Gearing RE, Schwalbe CS, Lee R and Hoagwood KE (2013) The effectiveness of booster sessions in CBT treatment for child and adolescent mood and anxiety disorders. Depression and Anxiety 30, 800–808. 23596102 10.1002/da.22118

[ref19] Guidi J, Brakemeier EL, Bockting CLH, Cosci F, Cuijpers P, Jarrett RB, Linden M, Marks I, Peretti CS, Rafanelli C, Rief W, Schneider S, Schnyder U, Sensky T, Tomba E, Vazquez C, Vieta E, Zipfel S, Wright JH and Fava GA (2018) Methodological recommendations for trials of psychological interventions. Psychotherapy and Psychosomatics 87, 276–284. 30007961 10.1159/000490574

[ref20] Hinton DE and Lewis-Fernández RC (2011) The cross-cultural validity of posttraumatic stress disorder: implications for DSM-5. Depression and Anxiety 28, 783–801. 21910185 10.1002/da.20753

[ref21] Jellinek MS, Murphy JM, Little M, Pagano ME, Comer DM and Kelleher KJ (1999) Use of the pediatric symptom checklist to screen for psychosocial problems in pediatric primary care: a national feasibility study. Archives of Pediatrics and Adolescent Medicine 153, 254–260. 10086402 10.1001/archpedi.153.3.254 PMC3905751

[ref22] Jordans MJD, Kohrt BA, Sangraula M, Turner EL, Wang X, Shrestha P, Ghimire R, Van't Hof E, Bryant RA, Dawson KS, Marahatta K, Luitel NP and van Ommeren M (2021) Effectiveness of group problem management plus, a brief psychological intervention for adults affected by humanitarian disasters in Nepal: a cluster randomized controlled trial. PLoS Medicine 18, e1003621. 34138875 10.1371/journal.pmed.1003621 PMC8211182

[ref23] Kessler RC, Andrews G, Colpe LJ, Hiripi E, Mroczek DK, Normand SLT, Walters EE and Zaslavsky AM (2002) Short screening scales to monitor population prevalences and trends in non-specific psychological distress. Psychological Medicine 32, 959–976. 12214795 10.1017/s0033291702006074

[ref24] Lakkis NA, Osman MH, Aoude LC, Maalouf CJ, Issa HG and Issa GM (2020) A pilot intervention to promote positive parenting in refugees from Syria in Lebanon and Jordan. Frontiers in Psychiatry 11, 257. 32372979 10.3389/fpsyt.2020.00257 PMC7179657

[ref25] Liddell BJ, O'Donnell M, Bryant RA, Murphy S, Byrow Y, Mau V, McMahon T, Benson G and Nickerson A (2021) The association between COVID-19 related stressors and mental health in refugees living in Australia. European Journal of Psychotraumatology 12, 1947564. 34434532 10.1080/20008198.2021.1947564 PMC8382014

[ref26] Lindsay G, Strand S and Davis H (2011) A comparison of the effectiveness of three parenting programmes in improving parenting skills, parent mental-well being and children's behaviour when implemented on a large scale in community settings in 18 English local authorities: the parenting early intervention pathfinder (PEIP). BMC Public Health 11, 962. 22208676 10.1186/1471-2458-11-962 PMC3316149

[ref27] Loewy RL, Pearson R, Vinogradov S, Bearden CE and Cannon TD (2011) Psychosis risk screening with the prodromal questionnaire--brief version (PQ-B). Schizophrenia Research 129, 42–46. 21511440 10.1016/j.schres.2011.03.029 PMC3113633

[ref28] Maguin E, Nochajski TH, De Wit DJ and Safyer A (2016) Examining the validity of the adapted Alabama parenting questionnaire-parent global report version. Psychological Assessment 28, 613–625. 26348028 10.1037/pas0000214 PMC4781672

[ref29] Mahfoud Z, Kobeissi L, Peters TJ, Araya R, Ghantous Z and Khoury B (2013) The Arabic validation of the Hopkins symptoms checklist-25 against MINI in a disadvantaged suburb of Beirut, Lebanon. International Journal of Educational and Psychological Assessment 13, 17–33.

[ref30] Miller KE, Koppenol-Gonzalez GV, Arnous M, Tossyeh F, Chen A, Nahas N and Jordans MJD (2020) Supporting Syrian families displaced by armed conflict: a pilot randomized controlled trial of the caregiver support intervention. Child Abuse and Neglect 106, 104512. 32408022 10.1016/j.chiabu.2020.104512

[ref31] Moitra M, Santomauro D, Collins PY, Vos T, Whiteford H, Saxena S and Ferrari AJ (2022) The global gap in treatment coverage for major depressive disorder in 84 countries from 2000–2019: a systematic review and Bayesian meta-regression analysis. PLoS Medicine 19, e1003901. 35167593 10.1371/journal.pmed.1003901 PMC8846511

[ref32] Nguyen TP, Guajardo MGU, Sahle BW, Renzaho AMN and Slewa-Younan S (2022) Prevalence of common mental disorders in adult Syrian refugees resettled in high income Western countries: a systematic review and meta-analysis. BMC Psychiatry 22, 15. 34986827 10.1186/s12888-021-03664-7 PMC8729124

[ref33] Norman GR, Sloan JA and Wyrwich KW (2003) Interpretation of changes in health-related quality of life: the remarkable universality of half a standard deviation. Medical Care 41, 582–592. 12719681 10.1097/01.MLR.0000062554.74615.4C

[ref34] Pan KY, Kok AAL, Eikelenboom M, Horsfall M, Jorg F, Luteijn RA, Rhebergen D, Oppen PV, Giltay EJ and Penninx B (2021) The mental health impact of the COVID-19 pandemic on people with and without depressive, anxiety, or obsessive-compulsive disorders: a longitudinal study of three Dutch case–control cohorts. The Lancet. Psychiatry 8, 121–129. 33306975 10.1016/S2215-0366(20)30491-0 PMC7831806

[ref35] Patel V, Weiss HA, Chowdhary N, Naik S, Pednekar S, Chatterjee S, De Silva MJ, Bhat B, Araya R, King M, Simon G, Verdeli H and Kirkwood BR (2010) Effectiveness of an intervention led by lay health counsellors for depressive and anxiety disorders in primary care in Goa, India (MANAS): a cluster randomised controlled trial. Lancet 376, 2086–2095. 21159375 10.1016/S0140-6736(10)61508-5 PMC4964905

[ref36] Patel V, Saxena S, Lund C, Thornicroft G, Baingana F, Bolton P, Chisholm D, Collins PY, Cooper JL, Eaton J, Herrman H, Herzallah MM, Huang Y, Jordans MJD, Kleinman A, Medina-Mora ME, Morgan E, Niaz U, Omigbodun O, Sondorp E, Pfaltz MC, Ruttenberg L, Schick M, Schnyder U, van Ommeren P, Ventevogel P, Weissbecker I, Weitz E, Wiedemann N, Whitney C and UnUtzer J (2018) The Lancet commission on global mental health and sustainable development. Lancet 392, 1553–1598. 30314863 10.1016/S0140-6736(18)31612-X

[ref37] Paykel ES (2007) Cognitive therapy in relapse prevention in depression. International Journal of Neuropsychopharmacology 10, 131–136. 16787553 10.1017/S1461145706006912

[ref38] Prigerson HG, Horowitz MJ, Jacobs SC, Parkes CM, Aslan M, Goodkin K, Raphael B, Marwit SJ, Wortman C, Neimeyer RA, Bonanno G, Block SD, Kissane D, Boelen P, Maercker A, Litz BT, Johnson JG, First MB and Maciejewski PK (2009) Prolonged grief disorder: psychometric validation of criteria proposed for DSM-V and ICD-11. PLoS Medicine 6, e1000121. 19652695 10.1371/journal.pmed.1000121 PMC2711304

[ref39] Rahman A, Hamdani SU, Awan NR, Bryant RA, Dawson KS, Khan MF, Azeemi MM, Akhtar P, Nazir H, Chiumento A, Sijbrandij M, Wang D, Farooq S and van Ommeren M (2016) Effect of a multicomponent behavioral intervention in adults impaired by psychological distress in a conflict-affected area of Pakistan: a randomized clinical trial. JAMA 316, 2609–2617. 27837602 10.1001/jama.2016.17165

[ref40] Rahman A, Khan MN, Hamdani SU, Chiumento A, Akhtar P, Nazir H, Nisar A, Masood A, Din IU, Khan NA, Bryant RA, Dawson KS, Sijbrandij M, Wang D and van Ommeren M (2019) Effectiveness of a brief group psychological intervention for women in a post-conflict setting in Pakistan: a cluster randomized controlled trial. Lancet 393, 1733–1744. 30948286 10.1016/S0140-6736(18)32343-2

[ref41] Riello M, Purgato M, Bove C, Tedeschi F, MacTaggart D, Barbui C and Rusconi E (2021) Effectiveness of self-help plus (SH + ) in reducing anxiety and post-traumatic symptomatology among care home workers during the COVID-19 pandemic: a randomized controlled trial. Royal Society of Open Science 8, 210219. 10.1098/rsos.210219 PMC8611343 34849238

[ref42] Shawyer F, Enticott JC, Block AA, Cheng IH and Meadows GN (2017) The mental health status of refugees and asylum seekers attending a refugee health clinic including comparisons with a matched sample of Australian-born residents. BMC Psychiatry 17, 76. 28222713 10.1186/s12888-017-1239-9 PMC5320723

[ref43] Shoeb M, Weinstein H and Mollica R (2007) The Harvard trauma questionnaire: adapting a cross-cultural instrument for measuring torture, trauma and posttraumatic stress disorder in Iraqi refugees. International Journal of Society Psychiatry 53, 447–463. 10.1177/0020764007078362 18018666

[ref44] Sijbrandij M, Acarturk C, Bird M, Bryant RA, Burchert S, Carswell K, de Jong J, Dinesen C, Dawson KS, El Chammay R, van Ittersum L, Jordans M, Knaevelsrud C, McDaid D, Miller K, Morina N, Park AL, Roberts B, van Son Y, … Cuijpers P (2017) Strengthening mental health care systems for Syrian refugees in Europe and the Middle East: integrating scalable psychological interventions in eight countries. European Journal of Psychotraumatology 8, 1388102. 29163867 10.1080/20008198.2017.1388102 PMC5687806

[ref45] Silove D, Sinnerbrink I, Field A, Manicavasagar V and Steel S (1997) Anxiety, depression and PTSD in asylum-seekers: associations with pre-migration trauma and post-migration stressors. British Journal of Psychiatry 170, 351–357. 10.1192/bjp.170.4.351 9246254

[ref46] Singla DR, Kohrt B, Murray LK, Anand A, Chorpita BF and Patel V (2017) Psychological treatments for the world: lessons from low-and middle-income countries. Annual Review of Clinical Psychology 13, 149–181. 10.1146/annurev-clinpsy-032816-045217 PMC5506549 28482687

[ref47] Thabet AA, Ibraheem AN, Shivram R, Winter EA and Vostanis P (2009) Parenting support and PTSD in children of a war zone. International Journal of Social Psychiatry 55, 226–237. 19383666 10.1177/0020764008096100

[ref48] WHODAS Group (2000) World Health Organisation Disability Assessment Achedule II. Available at https://www.who.int/standards/classifications/international-classification-of-functioning-disability-and-health/who-disability-assessment-schedule.

[ref49] World Health Organization (2016) Individual psychological help for adults impaired by distress in communities exposed to adversity. (Generic field-trial version 1.0). Available at http://www.who.int/mental_health/emergencies/problem_management_plus/en.

